# Heterologous Chimeric Construct Comprising a Modified Bacterial Superantigen and a Cruzipain Domain Confers Protection Against *Trypanosoma cruzi* Infection

**DOI:** 10.3389/fimmu.2020.01279

**Published:** 2020-06-30

**Authors:** María Belén Antonoglou, Andrés Sánchez Alberti, Daniela María Redolfi, Augusto Ernesto Bivona, María Julieta Fernández Lynch, Sofía Noli Truant, María Belén Sarratea, Laura Valeria Iannantuono López, Emilio Luis Malchiodi, Marisa Mariel Fernández

**Affiliations:** ^1^Cátedra de Inmunología, Departamento de Microbiología, Inmunología, Biotecnología y Genética, Facultad de Farmacia y Bioquímica, Universidad de Buenos Aires, Buenos Aires, Argentina; ^2^Instituto de Estudios de la Inmunidad Humoral “Prof. Ricardo A. Margni” (IDEHU), UBA-CONICET, Universidad de Buenos Aires, Buenos Aires, Argentina; ^3^Departamento de Microbiología, Parasitología e Inmunología, Facultad de Medicina and Instituto de Microbiología y Parasitología Médica (IMPaM), UBA-CONICET, Universidad de Buenos Aires, Buenos Aires, Argentina

**Keywords:** cruzipain, chimeric immunogen, immune modulators, *Trypanosoma cruzi*, mutant superantigen

## Abstract

Chagas disease is an endemic chronic parasitosis in Latin America affecting more than 7 million people. Around 100 million people are currently at risk of acquiring the infection; however, no effective vaccine has been developed yet. *Trypanosoma cruzi* is the etiological agent of this parasitosis and as an intracellular protozoan it can reside within different tissues, mainly muscle cells, evading host immunity and allowing progression towards the chronic stage of the disease. Considering this intracellular parasitism triggers strong cellular immunity that, besides being necessary to limit infection, is not sufficient to eradicate the parasite from tissues, a differential immune response is required and new strategies for vaccines against Chagas disease need to be explored. In this work, we designed, cloned and expressed a chimeric molecule, named NCz-SEGN24A, comprising a parasite antigen, the N-terminal domain of the major cysteine protease of *T. cruzi*, cruzipain (Nt-Cz), and a non-toxic form of the staphylococcal superantigen (SAg) G, SEG, with the residue Asn24 mutated to Ala (N24A). The mutant SAg SEGN24A, retains its ability to trigger classical activation of macrophages without inducing T cell apoptosis. To evaluate, as a proof of concept, the immunogenicity and efficacy of the chimeric immunogen vs. its individual antigens, C3H mice were immunized intramuscularly with NCz-SEGN24A co-adjuvanted with CpG-ODN, or the recombinant proteins Nt-Cz plus SEGN24A with the same adjuvant. Vaccinated mice significantly produced Nt-Cz-specific IgG titers after immunization and developed higher IgG2a than IgG1 titers. Specific cell-mediated immunity was assessed by *in-vivo* DTH and significant responses were obtained. To assess protection, mice were challenged with trypomastigotes of *T. cruzi*. Both schemes reduced the parasite load throughout the acute phase, but only mice immunized with NCz-SEGN24A showed significant differences against control; moreover, these mice maintained 100% survival. These results encourage testing mutated superantigens fused to specific antigens as immune modulators against pathogens.

## Introduction

Pathogens have evolved a diverse range of strategies to subvert the host immune system and survive. These strategies include evasion and modulation of the immune response, among others. Most of the intracellular parasites constitute a perfect example of this situation. *Trypanosoma cruzi* is the intracellular protozoan that causes Chagas disease, which affects 7 million people in America (1, 2) and leaves 100 million people in endemic areas at risk of acquiring the infection (3). The infection is contracted when the infective form of the parasite, the metacyclic trypomastigote, presents in the feces of hematophagous triatomines and penetrates a skin wound or an intact mucosa of the host. The acute phase of the disease exhibits non-specific symptoms, except for a periorbital eye inflammation denominated Romaña's sign, which usually occurs when the inoculation site is the conjunctiva. Although an important immune response is detected during this phase, it is inefficient to eradicate the parasite, allowing the development of the disease chronic period, which extends for decades. This stage is characterized by a low or undetectable parasitemia and most of the patients remain asymptomatic. After this period, around 30% of the infected people develop serious cardiac or digestive pathologies (4, 5). In addition to the worsening of the health, Chagas disease has also a high economic impact since it mostly affects economically active people (6). With the aim to develop an effective vaccine against *T. cruzi* to prevent or treat the infection, many antigens have been tested in preclinical studies in combination with different adjuvants [Reviewed in Cazorla et al. (7)]. In many of these studies, after experimental challenge vaccinated mice achieved 100% survival rates; however, clinical trials in humans were not performed with these formulations. In the last 20 years, recombinant proteins or DNA were employed as immunogens and, in combination with the latest generation of adjuvants, new vaccination strategies were developed with promising results [Reviewed in Bivona et al. (8)]. Cruzipain (Cz), the major cysteine protease of *T. cruzi*, has been studied by many authors as a fine antigen to evoke protective immune responses (9–16). In 2010, Cazorla et. al. described that the N-terminal domain of Cz (Nt-Cz) displayed the highest protective role during immunization, bypassing ineffective responses towards the C-terminal domain (17). In previous work from our laboratory, an engineered chimeric immunogen named Traspain was designed containing Nt-Cz as one of the key parasitic molecules, with very promising results (18, 19).

The development of effective prophylactic or therapeutic vaccines against *T. cruzi* requires the use of adjuvants and diverse delivery systems which promote a differential immune response. Bacterial Superantigens (SAgs) are enterotoxins which engage the T cell receptors (TCR) on the surface of T cells and the major histocompatibility complex (MHC) class II molecules on antigen presenting cells (APC) as non-processed molecules, eliciting an unregulated immune response characterized by a strong proinflammatory profile and a non-effective immune response. The idea of using this important immune modulatory characteristic of the SAgs for health care, gave rise to the construction of biotherapeutic tools for cancer treatment (20–22). Based on the capacity of SAgs to induce cross-presentation, modified SAgs were also used as adjuvants to improve the vaccination process (23).

The staphylococcal enterotoxin G (SEG) is a non-classical SAg which belongs to the group II or SEB family (24, 25). In the absence of T cells, SEG interacts with human monocytic/macrophagic cells inducing a strong M1 immune response with proinflammatory cytokines, which would eventually lead to a further T cell differentiation toward Th1/Th17 profiles (26). SAgs are also able to interact with dendritic cells without affecting their viability or migration to the secondary lymphoid organs. In a previous study (27), we demonstrated that SEG is phagocytized by murine dendritic cells, is found in sequential vesicles and afterwards released in the lymph node as a native and active molecule able to interact with TCR and MHC-II.

SAgs can stimulate T cells bearing certain variable β chain isoforms. The SAgs of group II are very well-characterized by their interaction with the mouse variable β chain 8.2 (mVβ). Classical SAgs, such as SEB and SEC3, bind the mVβ8.2 chain establishing three hydrogen bonds with residues located in the β chain through Asn24, with an affinity in the micromolar range. The affinity between SEG and mVβ8.2, which is in the nanomolar range, is the highest reported for staphylococcal superantigens (28). The complex SEG-Vβ8.2 has been crystallized, and the residues involved in the direct contact between these molecules are very well-documented (29). As it was also described for classical SAgs, the Asn24 on SEG surface is crucial for the interaction with the TCR and promotion of the T cell proliferation and further exhaustion (30).

These previous works prompted us to develop a mutant version of SEG, SEGN24A, which would be non-toxic but would maintain SAg's properties at innate level without exhaustion of the T cell responses. In addition, we designed, expressed, and tested as a proof of concept, a heterologous chimeric immunogen between the mutated bacterial SAg, SEGN24A, and the N-terminal domain of the major parasite antigen, Cz, with the aim to control *T. cruzi* infection in mice. The idea behind the design was that SEGN24A would contribute with its immune modulatory properties at the innate immune response, while Nt-Cz would confer specific protection to the immunized host. We demonstrated that the chimeric antigen, named NCz-SEGN24A adjuvanted with CpG-ODN, was able to protect against infection with *T. cruzi*, improving the performance of the administration of non-conjugated Nt-Cz and SEGN24A antigens.

## Materials and Methods

### Mice, Parasites and Cells

Different groups of inbred mice from the C3H/HeN strain were used in this work for immunization protocols. Mice breeding was carried out in the animal facilities of the “Instituto de Microbiología y Parasitología Médica” (IMPaM, UBA-CONICET). Experiments with animals were approved by the Review Board of Ethics of the School of Medicine (UBA, Argentina) and conducted in accordance with the guidelines established by the National Research Council (CONICET), CD resolution #: 3381-18.

*Trypanosoma cruzi* bloodstream trypomastigotes (from strains RA, Tulahuen-expressing β-galactosidase or K98 clone) were obtained from blood of CF1 infected mice.

Vero cells (ATCC CCL-81) were used for *T. cruzi in-vitro* infection and cultured in RPMI-1640 medium (Gibco) supplemented with 10% fetal bovine serum (FBS). RAW 264.7 cells (ATCC TIB-71) were used for evaluation of SAgs-mediated inhibition of proliferation and were cultured in DMEM medium (Hyclone) supplemented with 10% FBS. Both cell lines were regularly tested for *Mycoplasma* spp. infection with DAPI staining.

### Superantigens and Nt-Cz Recombinant Expression and Purification

Staphylococcal SEGwt was produced and purified as previously described (28). Briefly, SEGwt was cloned in pET-26b (Novagen, Merck), expressed in *Escherichia coli* BL21 (DE3) and purified by Ni^++^-NTA affinity chromatography (GE Healthcare), followed by *Superdex 200* molecular exclusion (GE Healthcare).

Hot spot punctual mutation on the TCR-binding site of SEGwt was introduced by site-directed mutagenesis (QuikChange™ Site-Directed Mutagenesis Kit, Stratagene). Asn24, which is essential for TCR binding and T cell activation (29), was replaced by Ala, generating the mutant SEGN24A. pET-26b plasmid encoding the *segn24a* gene was used to transform competent *E. coli* BL21 (DE3). SEGN24A protein was expressed in *E. coli* BL21 (DE3) as inclusion bodies, *in-vitro* refolded by drip dialysis method and furthered purified under native conditions by Ni^++^-NTA.

The N-terminal domain of Cz (Nt-Cz) was produced as previously described (17). Briefly, Nt-Cz was cloned in pET-23a, expressed in *Escherichia coli* BL21 (DE3) and purified by Ni^++^-NTA affinity chromatography, followed by *in vitro*-folding by dialysis. Purity levels of proteins were determined by SDS-PAGE.

Proteins were treated with agarose-polymixin B (Sigma Aldrich) to remove endotoxin traces and absence of LPS was assessed by Limulus test. In addition, the amount of remaining endotoxin was evaluated by the Pierce LAL chromogenic endotoxin quantitation Kit (Thermo Scientific) following the manufacturers' instructions. For all recombinant proteins, it was verified that <0.1 EU/ml of endotoxin was present.

### Flow Cytometry for *in-vivo* Stimulation of T Cells Bearing TCR Vβ8.1/8.2

Female C3H/HeN mice 6 to 8 weeks-old (*n* = 3/group) were inoculated subcutaneously into the footpad with 30 μg of SEGwt or SEGN24A, or sterile PBS (control). After 48 h, mice were sacrificed, and their popliteal and inguinal lymph nodes were removed. Single cell suspensions were obtained from each node by mechanical dislodging. Cells were immunolabeled with e-Fluor 660-conjugated anti-mouse CD3 antibody (e-Bioscience Inc.) and FITC-conjugated anti- Vβ8.1/8.2 TCR (e-Bioscience, Inc.). To perform suitable data acquisition and analysis, autofluorescence and single-stained controls were included. Stained cells were passed through the BD FACSCanto flow cytometer. Data were analyzed using the FlowJo v10 software (Tree Star, Inc.).

### Surface Plasmon Resonance Assay (SPR)

SPR assays were performed using a BIAcore T100 instrument (GE Healthcare). SEGwt or SEGN24A (ligands) were immobilized on a CM5 chip (GE Healthcare) surface by amine coupling according to the manufacturer's instructions. Soluble TCR β chain Vβ8.2 protein (analyte) was diluted in PBS, pH 7.4 running buffer, and injected over chip surfaces at a flow rate of 30 μl × min^−1^ for 60 s at 25°C. The data were analyzed with the BIA evaluation software (GE Healthcare). To avoid conformational restrictions due to the immobilization procedure, the assay was also performed immobilizing TCR β chain Vβ8.2 over the chip surface and using SEGwt or SEGN24A as analyte (31).

### Cell Inhibition Assays

To evaluate the ability of SAgs to inhibit macrophage proliferation, RAW 264.7 cells (2 × 10^4^/well) were cultured in the presence of SEGwt, SEGN24A (10 μg/ml) or medium (control) for 48 h at 37°C in 5% (v/v) CO_2_. During the last 8 h, 0.5 μCi [^3^H]-thymidine/well was added and then harvested onto glass fiber filters. Incorporation of radioactivity was then measured using a Liquid Scintillation Analyzer Tri–Carb 2810 TR (Perkin Elmer). All measurements were made in triplicate.

### Nitrite Assay

Nitrites production was evaluated in culture supernatants by Griess reaction. Briefly, RAW 264.7 cells (1 × 10^5^/well) were cultured in the presence of SEGwt, SEGN24A (10 μg/ml) or medium (control). At 48 h supernatants were recovered, mixed with same volume of A + B Griess reagent (Laboratorios Britania) and then incubated in the dark for 10 min at room temperature.

The absorbance of the reaction mixture was measured at 540 nm and the nitrites concentration in the samples was determined using a sodium nitrite (NaNO_2_) standard curve.

### NCz-SEGN24A Construction, Recombinant Expression and Characterization

The heterologous chimeric *ncz-segn24a* gene was constructed using splicing by overlap extension PCR (SOE-PCR) (32, 33). Supplementary Figure 1 provides details on the PCRs that were performed in order to fuse *nt-cz* and *segn24a* genes. The resultant fused chimeric gene was *ncz-segn24a*. *Bam*HI restriction site was included in PF_1_, and PR_4_ included *Eco*RI restriction site as well as a 6 his tag coding sequence. Cloning was done in a pET-32a(+) vector, which allowed fusion to thioredoxin (Trx). Gene sequencing was performed to confirm the chimeric gene.

NCz-SEGN24A (MW 69 kDa) protein was expressed in *E. coli* BL21 (DE3) as inclusion bodies, purified under denaturing conditions by Ni^++^-NTA, *in-vitro* refolded by dialysis method and furthered purified by *Superdex 200* molecular exclusion (GE Healthcare). Purity levels were determined by SDS-PAGE. LPS absence was determined by the Pierce LAL chromogenic endotoxin quantitation Kit (Thermo Scientific) following the manufacturers' instructions.

Immunoblotting was performed in order to verify NCz-SEGN24A identity. Briefly, 10 μg of NCz-SEGN24A, Nt-Cz (MW 23 kDa) and SEGN24A (MW 27 kDa) were separated by 12.5% SDS-PAGE and transferred to polyvinylidene difluoride (PVDF) membrane. As primary antibodies, sera from mice immunized with the recombinant protein Nt-Cz or SEG superantigen were used (1/500 dilution). As secondary antibody, rabbit anti-mouse IgG conjugated with peroxidase (Jackson ImmunoResearch, 1/1000 dilution) was used; the reaction was revealed using 4-chloro-1-naphtol and H_2_O_2_.

### Immunization Protocols

As a proof of concept, different immunization protocols were tested in this work. Inbred C3H/HeN mice from 6 to 8 weeks old (*n* = 5-6 animals per group) were used. In all cases, animals were immunized with 4 doses of each formulation separated by 15 days, intramuscularly in the quadriceps muscles.

To initially prove properties of NCz-SEGN24A as an immunogen, two experimental groups were designed: (i) 10 μg of recombinant protein NCz-SEGN24A and (ii) control with PBS (control group in each protocol).

Secondly, the chimeric protein was proved in parallel with its individual antigens, using the adjuvant CpG-ODN 1826 (Invivogen). In this case, three experimental groups were designed: (i) NCz-SEGN24A protein+CpG, (ii) Nt-Cz protein+SEGN24A+CpG, (iii) control. Each group received 10 μg of each component, with the exception of group (ii) that received 5 μg of each antigen.

In the last part of this work, NCz-SEGN24A was evaluated against the single *T. cruzi* specific antigen; in order to achieve this, three experimental groups were immunized: (i) NCz-SEGN24A protein+CpG, (ii) Nt-Cz protein+CpG, (iii) control. Each group received 10 μg of each component.

### ELISA for Determination of Antigen-Specific IgG Antibodies

Sera from immunized mice were collected 15 days after the last dose for the determination of antigen-specific IgG, IgG1, IgG2a by indirect ELISA, as previously described (34). Plates were coated with Nt-Cz protein in all cases, and horseradish peroxidase-labeled anti-mouse IgG antibody (Jackson ImmunoResearch), or biotin-conjugated anti-mouse IgG1 or anti-mouse IgG2a (Pharmingen Becton Dickinson), were used as secondary antibodies.

### Cell Infection Inhibition by Specific Immune Sera

The ability of sera from immunized mice to inhibit parasite infection was assessed. Vero cells (5 × 10^3^ cells/well) were infected with blood trypomastigotes expressing β-galactosidase at a MOI of 10:1 for 24 h at 37°C. Trypomastigotes were pre-incubated in triplicate with diluted serum (1/10) from mice immunized with NCz-SEGN24A+CpG. After overnight incubation, cells were washed and treated for 4 days. CPRG was added to determine the levels of parasites as previously described (35). Uninfected cells were used as blanks, and additional controls were performed with sera from mice before immunization (pre-immune) and sera from non-immunized mice.

### Delayed-Type Hypersensitivity Test (DTH)

A DTH assay was performed 15 days after the last immunization by intradermal challenge with 10 μg of Nt-Cz in the right footpad of the animals. Footpad thickness was measured before and 72 h after antigen inoculation using a Vernier caliper. Results are expressed as the increased millimeters in footpad thickness induced by inoculation.

### Challenge With *T. cruzi* Bloodstream Trypomastigotes

Fifteen days after the last immunization, animals (*n* = 5-6 per group) were infected intraperitoneally with 10^4^ *T. cruzi* blood trypomastigotes of the virulent RA strain, for analysis of the acute phase. Parasitemia was monitored by counting blood parasites every 2–3 days in a Neubauer chamber. For this purpose, a 1/5 dilution of blood in lysis buffer (0.75% NH_4_Cl, 0.2% Tris, pH 7.2) was made.

Alternatively, vaccine efficacy was evaluated in a sub-lethal challenge. For this purpose, after the last immunization animals were challenged with 3 × 10^5^ blood trypomastigotes of the less virulent K98 clone (from CA-I strain) by intraperitoneal injection. Parasitemias were recorded weekly during the acute phase (19, 35).

### Statistical Analysis

Statistical analysis was carried out with GraphPad Prism 6.0 software (San Diego, CA, USA), using one-way or two-way ANOVA along with the post-tests indicated in each trial, unless otherwise specified in figure legends.

The statistical analyses were referred to the control group of each experiment, except when indicated. Values of *p* < 0.05 were considered significant.

## Results

### Mutant SEGN24A Neither Binds Mouse TCR Variable β Chain nor Stimulates *in-vivo* Vβ8.1/8.2 Bearing T Cells

**Figure 1:**
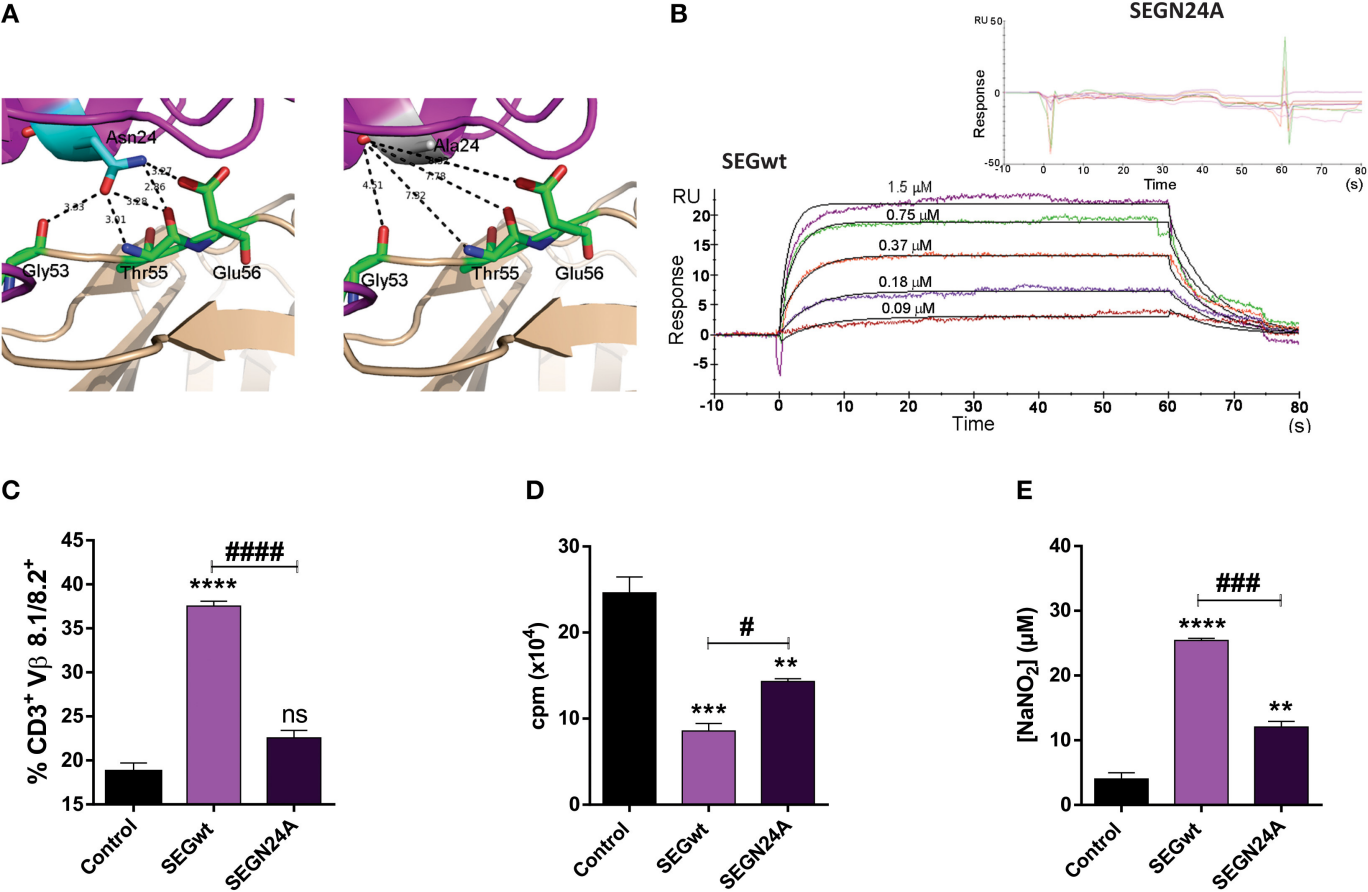
Superantigen mutant SEGN24A shows no effect on T cells but retains ability to induce a proinflammatory profile in RAW 264.7 macrophages. **(A)** Comparison of the TCR mVβ8.2 and SEGwt (pdb 1XXG) or SEGN24A interfaces. mVβ8.2 and SEG are colored wheat and magenta, respectively. Residues of mVβ8.2 involved in the interaction with residue 24 of SEGwt or SEGN24A are colored green. SEG N24 (left side) or A24 (right side) are colored cyan or white, respectively. Hydrogen bonds established between Asn24 and mVβ8.2 are shown as black dashes. Since hydrogen bonds must be calculated with a cut-off distance of 2.5–3.4 Å, Ala24 is not able to establish hydrogen bonds with mVβ8.2. **(B)** SPR analysis of SEG-mVβ8.2 interaction. SPR sensorgrams show the interactions between SEGwt (800 RU immobilized, lower panel) with mVβ8.2 (1.50–0.09 μM), or SEGN24A (900 RU immobilized, upper panel) with mVβ8.2 (60–0.8 μM) after correction for nonspecific binding. Apparent K_D_ for binding of mVβ8.2 to immobilized SEGwt was 3.7 × 10^−7^ M by kinetic analysis using a 1:1 binding model. SEGN24A showed no specific binding to mVβ8.2. **(C)** C3H/HeN mice were inoculated subcutaneously with 30 μg of SEGwt, SEGN24A or PBS (control) into the mice footpad. After 48 h, cells were isolated from popliteal and inguinal lymph nodes and T cell stimulation was evaluated by flow cytometry with anti-CD3^+^ and anti-Vβ8.1/8.2^+^. **(D)** RAW 264.7 cells (2 × 10^4^/well) were incubated with wild-type or mutant superantigens (10 μg/ml) for 48 h, 0.5 μCi [3H]-thymidine/well was added for the last 8 h. DNA was harvested and counts per minute were determined as a measure of cell proliferation. **(E)** Supernatants of RAW 264.7 cells (1 × 10^5^/well) were collected after 48 h of stimulation with SAgs and the nitrites level was measured using the Griess reagent. Data are expressed as the mean ± SEM of three independent experiments. Asterisks represent statistical significance respect to control. **p < 0.01, ***p < 0.001, ****p < 0.0001, ns, non-significant. Hashes represent statistical significance between stimulation with wild-type and mutant SAg, ^#^*p* < 0.05, ^###^*p* < 0.001, ^####^*p* < 0.0001. One-way ANOVA plus Tukey's post-test **(C–E)**.

Taking into account the high resolution X-ray structure of SEGwt-mVβ8.2 (pdb 3MC0) as a template (29), selective mutation of Asn24, crucial residue for binding to the TCR in related SAgs (30), resulted in the generation of SEGN24A. Structural studies strongly suggested that higher affinity of SEG-mVβ8.2 complex is due to the presence of five hydrogen bonds established between SEGN24 and different residues of the TCR variable β chain. Due to the nature of the Ala side chain, hydrogen bonds cannot be established with the TCR residues since they are outside of the interaction sphere (distances higher than 3.4 Å) (Figure 1A). In order to evaluate the resultant ability of mutant SEGN24A to bind mVβ8.2 chain, surface plasmon resonance (SPR) assays were performed. Direct binding of soluble SEGwt or SEGN24A to mVβ8.2 was analyzed. SEGN24A failed to bind mVβ8.2 specifically. On the contrary, the interaction between SEGwt and mVβ8.2 showed a high affinity with a dissociation constant (K_D_) of 3.7 × 10^−7^ M (Figure 1B), in accordance with our previous results (29).

We next analyzed whether SEGN24A would be able to induce stimulation of T cells bearing Vβ8.1/8.2 *in-vivo*. SEGwt and SEGN24A were subcutaneously injected into C3H mice footpad. After 48 h, mice were sacrificed, and their popliteal and inguinal lymph nodes removed and pooled for analysis of Vβ8.1/8.2 T cell populations. FACS analysis showed a significant increase in the frequency of Vβ8.1/8.2^+^ T cells in lymph nodes stimulated with SEGwt, while SEGN24A was unable to stimulate Vβ8.1/8.2^+^ T cells (Figure 1C).

### SEGN24A Retains, in a Lesser Extent, SEGwt Effects on Macrophages

To verify if SEGN24A retained the ability to induce a M1 profile on macrophages, a mouse macrophagic cell line RAW 264.7 was cultured in presence of SEGN24A or SEGwt. Although in a lesser extent, the mutated SAg was able to mimic the effect of SEGwt. Both wild type and mutant SAgs induced an inhibition of macrophagic proliferation in absence of T cells (Figure 1D). This phenomenon was previously related to apoptotic and necrotic death with an early activation and secretion of proinflammatory cytokines (26). The cell death process would be of interest considering that other adjuvants such as aluminum salts mediate their adjuvant-properties in part through the release of damage associated molecular pattern (DAMPs) after exerting cytotoxicity (36, 37). Moreover, SEGN24A induced a significant increment in nitrites production (Figure 1E), characteristic of classical macrophage activation.

These results confirm that while SEGN24A has no TCR binding function (Figure 1B), it keeps the ability to interact with macrophagic cells, an essential feature to exert an adjuvant effect.

### Construction, Expression and Characterization of the Heterologous Chimeric Antigen NCz-SEGN24A

**Figure 2:**
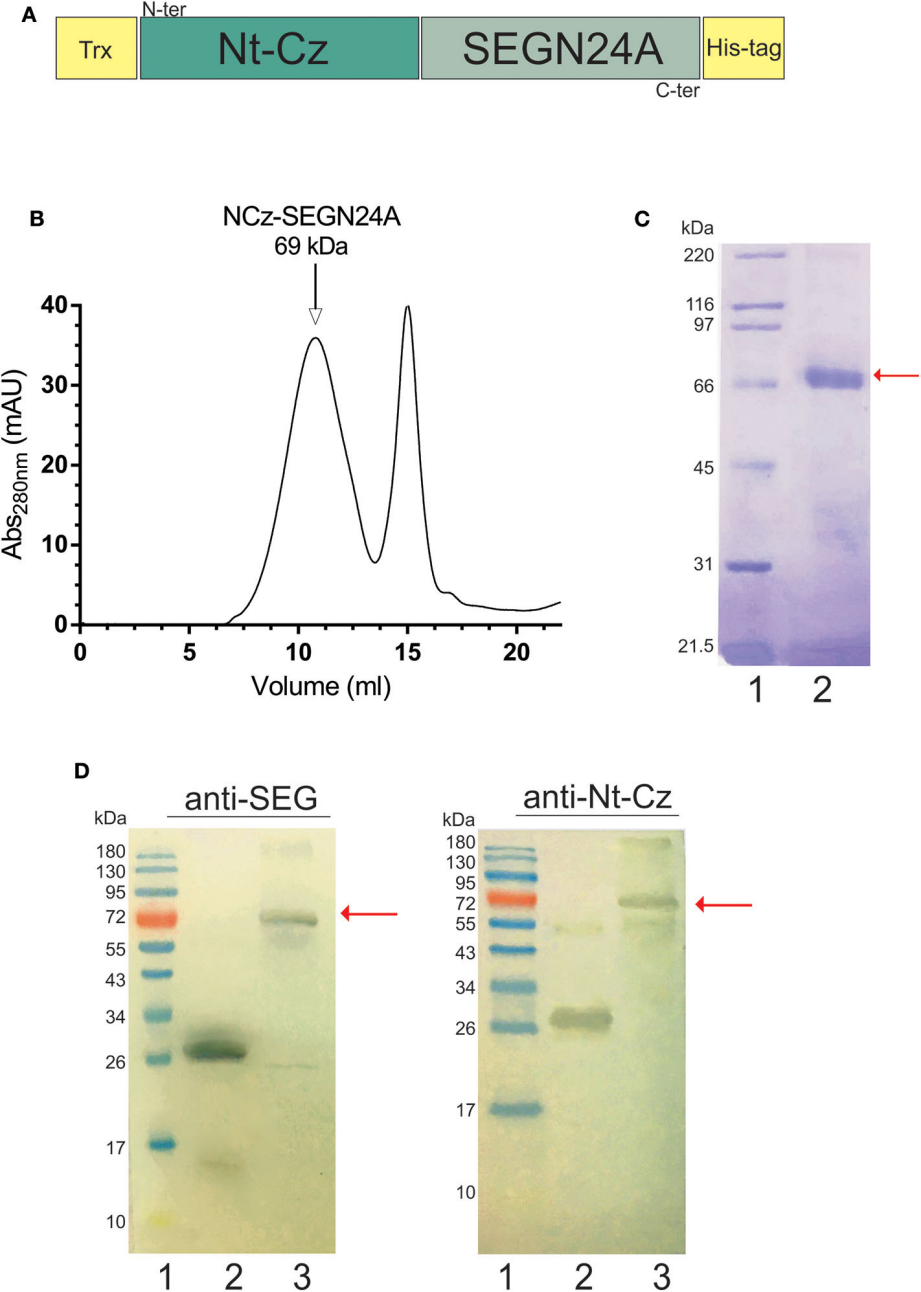
Construction and characterization of heterologous chimeric antigen NCz-SEGN24A. **(A)** Schematic representation of NCz-SEGN24A. Bacterial SAg mutant SEGN24A was fused to the C-terminal of the N-terminal domain of parasite cruzipain (Nt-Cz). A thioredoxin (Trx) tag was added to fused NCz-SEGN24A in its N-terminal, and histidine tag (His-tag) was added at the C-terminal. **(B)** Chromatogram of the NCz-SEGN24A purification with Superdex 200 molecular exclusion (SEC). **(C)** Analysis of recombinant NCz-SEGN24A by SDS-PAGE. The 69-kDa protein was detected using Coomassie blue staining after purification by Ni^2+^-NTA column followed by SEC (Lane 2). Arrow indicates NCz-SEGN24A band. **(D)** Immunochemical identity by Western blot. Domain-specific polyclonal antibodies were used as primary antibodies. SDS-PAGE gels were loaded as follows, lines: 1- MWM, 2- SEGN24A (left, 27 kDa) or Nt-Cz (right, 23 kDa), 3- NCz-SEGN24A (69 kDa), arrows point at NCz-SEGN24A band.

The heterologous chimera between a parasite antigen domain and a bacterial mutated superantigen, NCz-SEGN24A, was engineered fusing the Nt-Cz domain directly to the mutant SEGN24A. The construct also possesses a thioredoxin N-terminal tag, to facilitate folding and stability due to disulfide bonds formation, and a 6 his tag to allow purification (Figure 2A). NCz-SEGN24A was successfully expressed in *E. coli* BL21 (DE3) and purified by Ni^2+^-NTA affinity chromatography. Refolding of recombinant NCz-SEGN24A was performed *in-vitro*, followed by molecular exclusion chromatography (Figure 2B). A single 69 kDa band was obtained by SDS-PAGE (Figure 2C). Final yield of NCz-SEGN24A protein was ~5 mg per liter of culture.

The immunochemical identity of the construct was furthered determined by Western Blot. NCz-SEGN24A was recognized by polyclonal antibodies specific for both domains (Figure 2D).

### Immunization With NCz-SEGN24A Elicits a Humoral and Cellular Specific Immune Response

**Figure 3:**
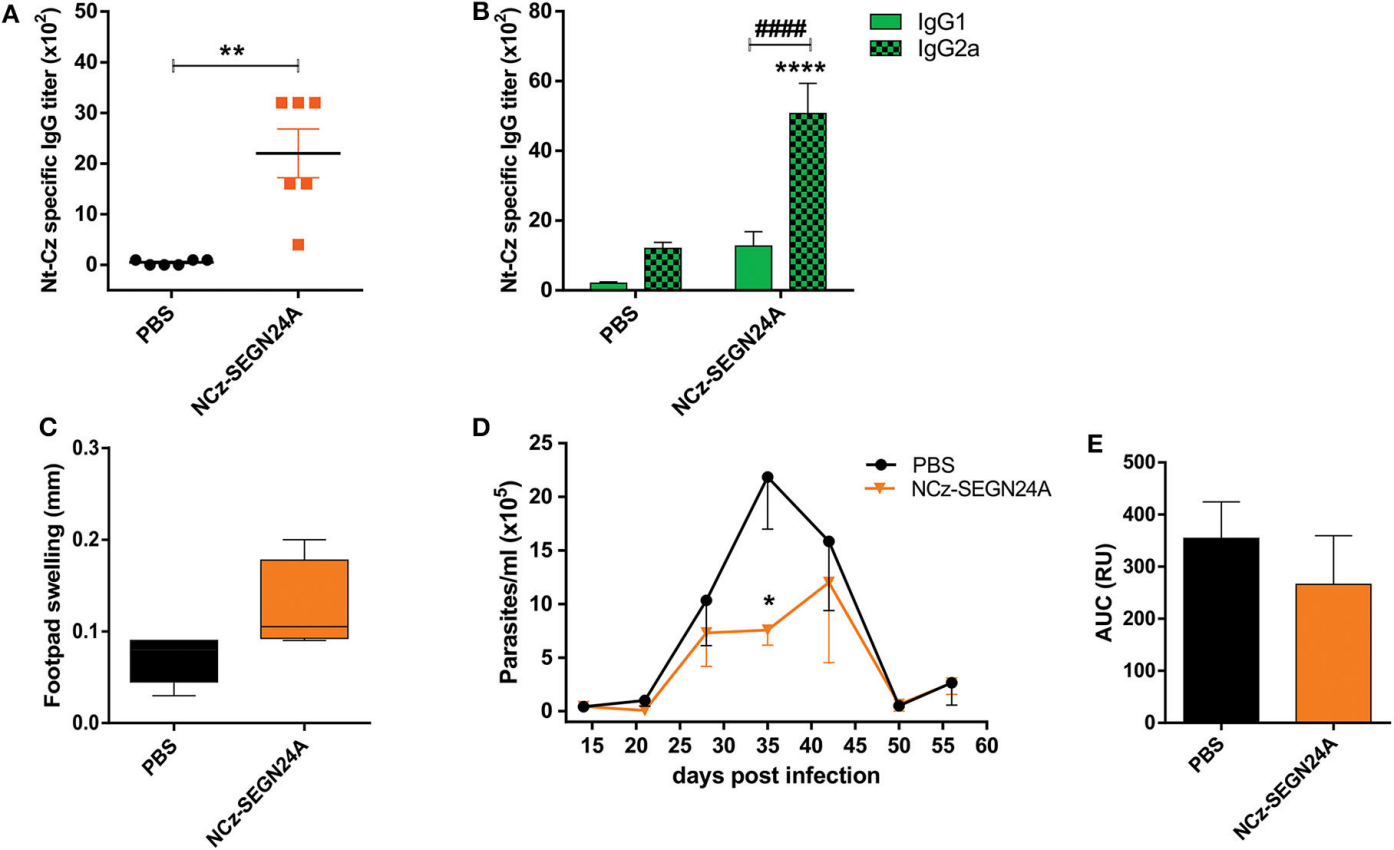
NCz-SEGN24A develops a strong and Th1-directed humoral response and decreases parasite loads after *T. cruzi* challenge. **(A)** Nt-Cz-specific IgG antibodies response. Titers were determined by ELISA in sera samples from C3H/HeN mice vaccinated with either NCz-SEGN24A, by the intramuscular route, or saline (PBS) at 15 days post vaccination. **(B)** Nt-Cz specific IgG1 and IgG2a titers, determined by indirect ELISA using an isotype-specific secondary antibody. **(C)** Delayed-type hypersensitivity (DTH) test. Footpad thickness in vaccinated mice was measured before and 72 h after inoculation of 10 μg of Nt-Cz. Results are expressed as the difference in footpad thickness before and after inoculation. **(D)** Protection against a *T. cruzi* challenge. Fifteen days after the last immunization, mice were challenged with 3 × 10^5^ K98 clone bloodstream trypomastigotes. Parasitemia after infection was monitored weekly during its acute phase. **(E)** Area under the parasitemia curve (AUC). Data are expressed as the mean ± SEM of two independent experiments. Asterisks represent statistical significance respect to saline group: *p < 0.05; **p < 0.01; ****p < 0.0001. Hashes represent statistical significance between IgG1 and IgG2 isotypes: ^####^*p* < 0.0001. Mann-Whitney test **(A)**, two-way ANOVA plus Sidak's post-test **(B)**, Student's *t*-test **(C,E)**.

With the aim to evaluate the immunogenicity of adjuvantless NCz-SEGN24A, C3H mice were immunized with four doses of the recombinant protein and 15 days after last boost, Nt-Cz-specific antibody titers in sera were determined by ELISA. We observed that mice immunized with NCz-SEGN24A elicited antibody titers considerably higher than control group (*p* < 0.01) (Figure 3A). To estimate the developed T cell profile, we analyzed the titers of the IgG1 and IgG2a isotypes specific against Nt-Cz. Antibodies isotypes reflected a Th1-biased response since IgG2a levels were higher than IgG1 (*p* < 0.0001) (Figure 3B).

A delayed-type hypersensitivity test was performed to evaluate the specific cellular immune response. Mice developed a tendency of swelling regarding a cellular response, 72 h after the injection of Nt-Cz in the footpad (0.13 ± 0.03 for NCz-SEGN24A vs. 0.07 ± 0.01 for control) (Figure 3C).

These results were encouraging since a parasite homologous chimeric immunogen, Traspain (18), was not able to promote specific humoral or cellular immune responses (Sanchez Alberti, unpublished results).

### NCz-SEGN24A Immunization Displays a Tendency of Parasite Load Reduction After *T. cruzi* Challenge

To evaluate if immunization with NCz-SEGN24A was able to confer protection, 15 days after last dose mice were challenged with bloodstream trypomastigotes of the K98 clone, from *T. cruzi* CA-I strain. As it is shown in Figure 3D, immunization with NCz-SEGN24A resulted in a reduction of the number of circulating parasites. Furthermore, at the peak of parasitemia (35 dpi) there was a significant reduction of parasites compared against control (PBS) (p < 0.05). We also calculated the area under the parasitemia concentration-time curve (AUC) to assess the ability of the vaccination to reduce the total parasite load. In this case, we observed a reduction of the total parasite load conferred by immunization with NCz-SEGN24A, although there was not a significant difference compared to control (AUC: 112.5 ± 36.9 vs. 132.8 ± 41.0, respectively) (Figure 3E).

### Co-administration of NCz-SEGN24A With CpG-ODN Evokes Specific Humoral Responses and Develops Neutralizing Antibodies

**Figure 4:**
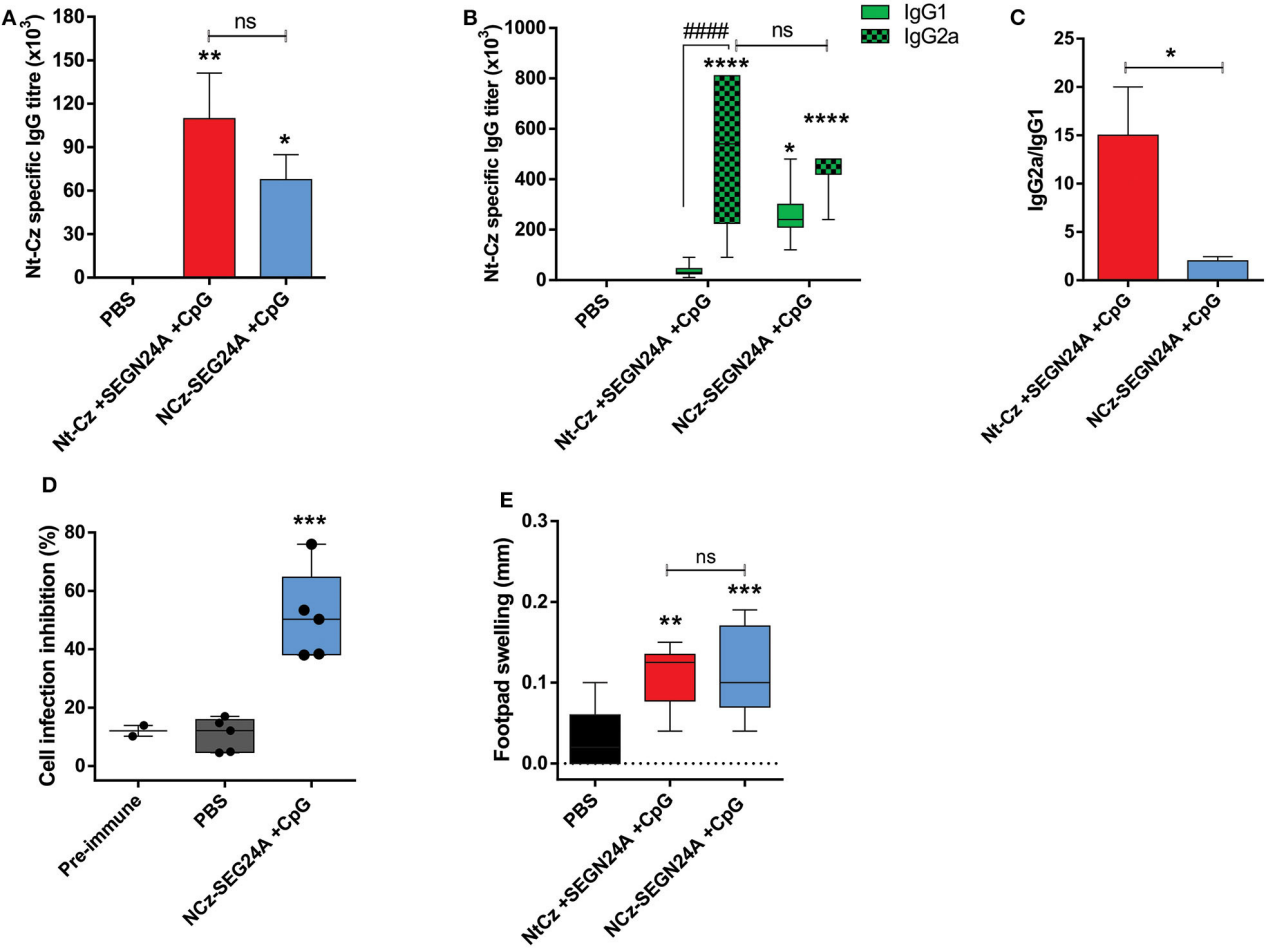
Co-administration of NCz-SEGN24A with CpG-ODN evokes specific humoral responses and develops neutralizing antibodies. Mice were either immunized with chimeric protein NCz-SEGN24A+CpG or with individual antigens Nt-Cz+SEGN24A+CpG intramuscularly, or saline. **(A)** Nt-Cz specific IgG antibodies determined by ELISA in sera samples, 15 days after last immunization. **(B)** Nt-Cz specific IgG1 and IgG2a isotypes titers, determined by ELISA. **(C)** IgG2a/IgG1 ratio **(D)** Neutralization of *T. cruzi* non-phagocytic cell infection by sera from immunized mice. **(E)** DTH test, results are expressed as the difference in footpad thickness before and after 72 h inoculation with Nt-Cz antigen. Data are expressed as the mean ± SEM of three independent experiments. Asterisks represent statistical significance respect to saline group, except indicated otherwise: *p < 0.05, **p < 0.01, ***p < 0.001, ****p < 0.0001, ns, non-significant difference. Hashes represent statistical significance between IgG1 and IgG2 isotypes: ^####^*p* < 0.000. Kruskal–Wallis plus Dunn's post-test **(A)**, two-way ANOVA plus Sidak's post-test **(B)**, one-way ANOVA plus Tukey's post-test **(C,D)**.

Taking into account the well-known capacity of CpG-ODN to stimulate Th1-responses (38, 39), we combined them with the chimeric NCz-SEGN24A. Considering that the protective effect could be due to the administration of one molecule that would function as an immune modulator and another that would serve as the specific immunogenic antigen (40, 41), we also developed an immunization protocol that included the individual proteins Nt-Cz and SEGN24A co-administered with CpG-ODN. Immunization groups through the intramuscular route included: Nt-Cz+SEGN24A+CpG, or the chimeric NCz-SEGN24A+CpG, all the antigens in equal molar mass. Analysis of the specific antibody response obtained after the last dose showed that both groups developed high and significant IgG anti-Nt-Cz specific titers (*p* < 0.01 and *p* < 0.05, respectively), which did not differ from each other (Figure 4A). Interestingly, immunization with both, the individual antigens and the chimeric antigen plus CpG-ODN elicited significant IgG2a specific antibodies, reflecting a Th1-biased response (Figure 4B). We observed an unbalanced ratio IgG2a/IgG1 for the immunization with the separated proteins, suggesting a more balanced immunity when NCz-SEGN24A plus CpG-ODN is used as immunogen (Figure 4C). As expected, antibodies specific titers resulted about 10-fold higher when immunization was performed with CpG-ODN compared to adjuvantless injection (Figure 3A).

More importantly, Nt-Cz-specific antibodies proved to be functional and neutralizing, since incubation of trypomastigotes of Tulahuen strain with serum of NCz-SEGN24A-vaccinated mice significantly decreased *in-vitro* invasion of non-phagocytic Vero cells (Figure 4D).

Moreover, when analyzing cellular *in-vivo* responses, a significant increase in DTH assay was observed for both immunization with individual proteins or NCz-SEGN24A (*p* < 0.01 and *p* < 0.001, respectively) (Figure 4E).

### NCz-SEGN24A Plus CpG-ODN but Not Its Individual Antigens Confer Protection Against *T. cruzi* Challenge

**Figure 5:**
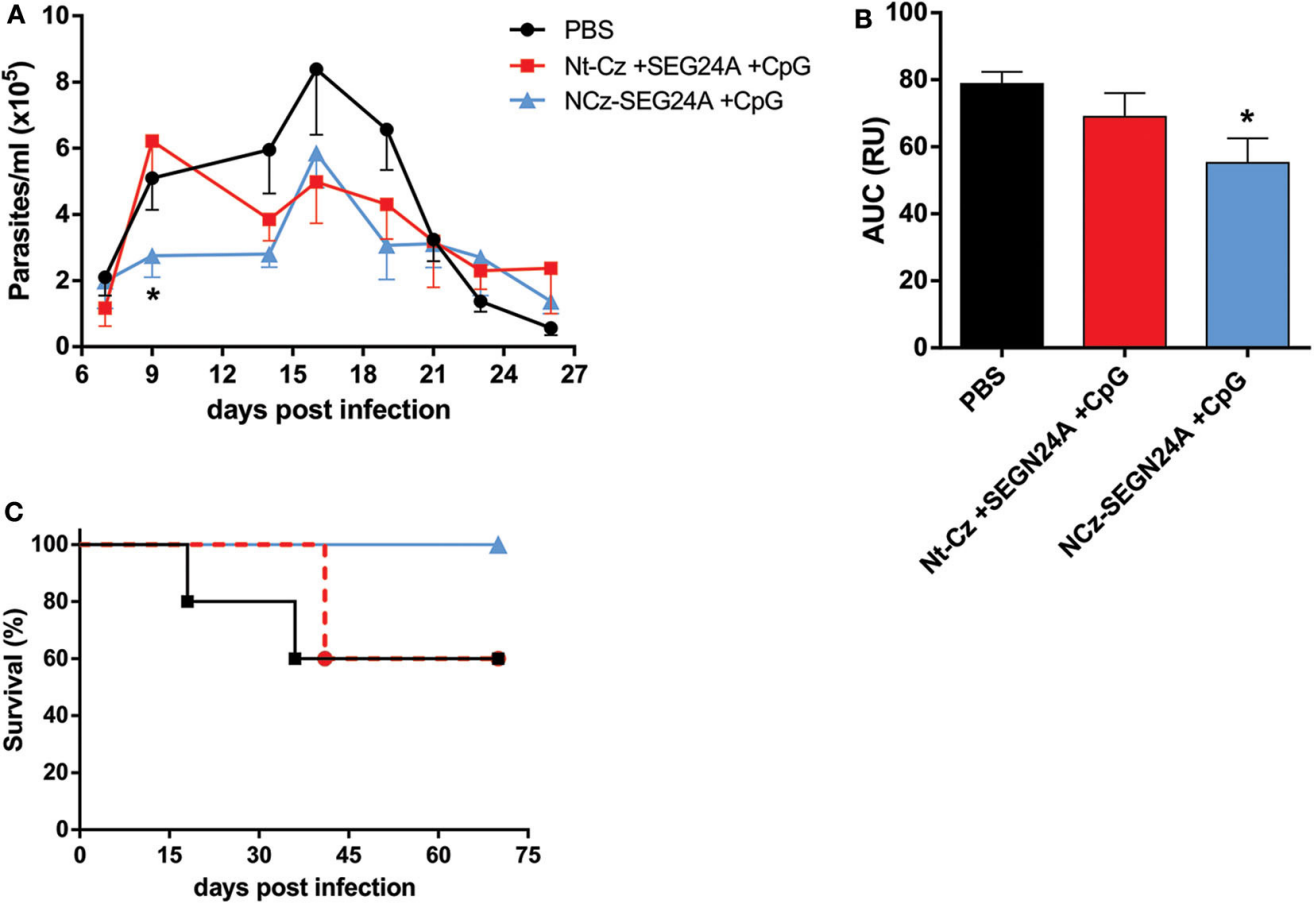
NCz-SEGN24A+CpG, but not its individual antigens, confers protection against *T. cruzi* infection. Immunized mice with NCz-SEGN24A+CpG, individual antigens Nt-Cz+SEGN24A+CpG or saline (*n* = 5/group) were challenged intraperitoneally 15 days after the last dose with 1 × 10^4^ trypomastigotes from the RA strain. **(A)** Parasitemia levels were monitored during the acute phase of the infection in 5 μl of blood taken every 2 days. **(B)** AUC of the parasitemia curve. **(C)** Survival rates were monitored daily over an extended period of the acute phase. Data are expressed as the mean ± SEM of three independent experiments. Asterisks represent statistical significance respect to saline group. *p < 0.05. One-way ANOVA plus Tukey's post-test **(B)**.

To determine whether chimeric NCz-SEGN24A adjuvanted with CpG-ODN possessed superior protective capacity against *T. cruzi* infection compared to its individual domains, 2 weeks after immunization mice were challenged with blood trypomastigotes from the virulent RA strain, belonging to the discrete type unit (DTU) TcVI. Throughout the acute phase of the parasitemia, NCz-SEGN24A+CpG showed the ability to reduce the level of circulating parasites (Figure 5A). We observed that on the early acute phase (9 dpi) NCz-SEGN24A+CpG group achieved the highest control of parasitemia. Moreover, when the area under the parasite-load curves were analyzed (Figure 5B), NCz-SEGN24A+CpG showed a significant reduction on the total parasite load compared with the control group, whereas the individual antigens Nt-Cz plus SEGN24A+CpG conferred less protection.

More importantly, mice vaccinated with chimeric NCz-SEGN24A+CpG showed an increase in the survival rate compared to PBS group. Specifically, this group was the only one to maintain 100% survival rates, showing clear differences against the group of mice immunized with the individual antigens (60% survival for both Nt-Cz+SEGN24A+CpG and control) (Figure 5C).

These results taken together manifest a clear advantage of administration of the chimeric NCz-SEGN24A plus CpG-ODN rather than immunizing with its individual domains, in terms of protection and survival against infection with *T. cruzi*.

### Chimeric NCz-SEGN24A Improves Protection Elicited by Nt-Cz Antigen

As it was previously reported, immunization of mice with Nt-Cz plus CpG-ODN enhances protection conferred by vaccination with the full-length cruzipain or its C-terminal domain (17). To evaluate if vaccination with chimeric NCz-SEGN24A antigen would improve protection over the single *T. cruzi* antigen, we performed an immunization protocol where a group of mice received Nt-Cz+CpG, and other group received NCz-SEGN24A+CpG (10 μg of each in both schemes).

**Figure 6:**
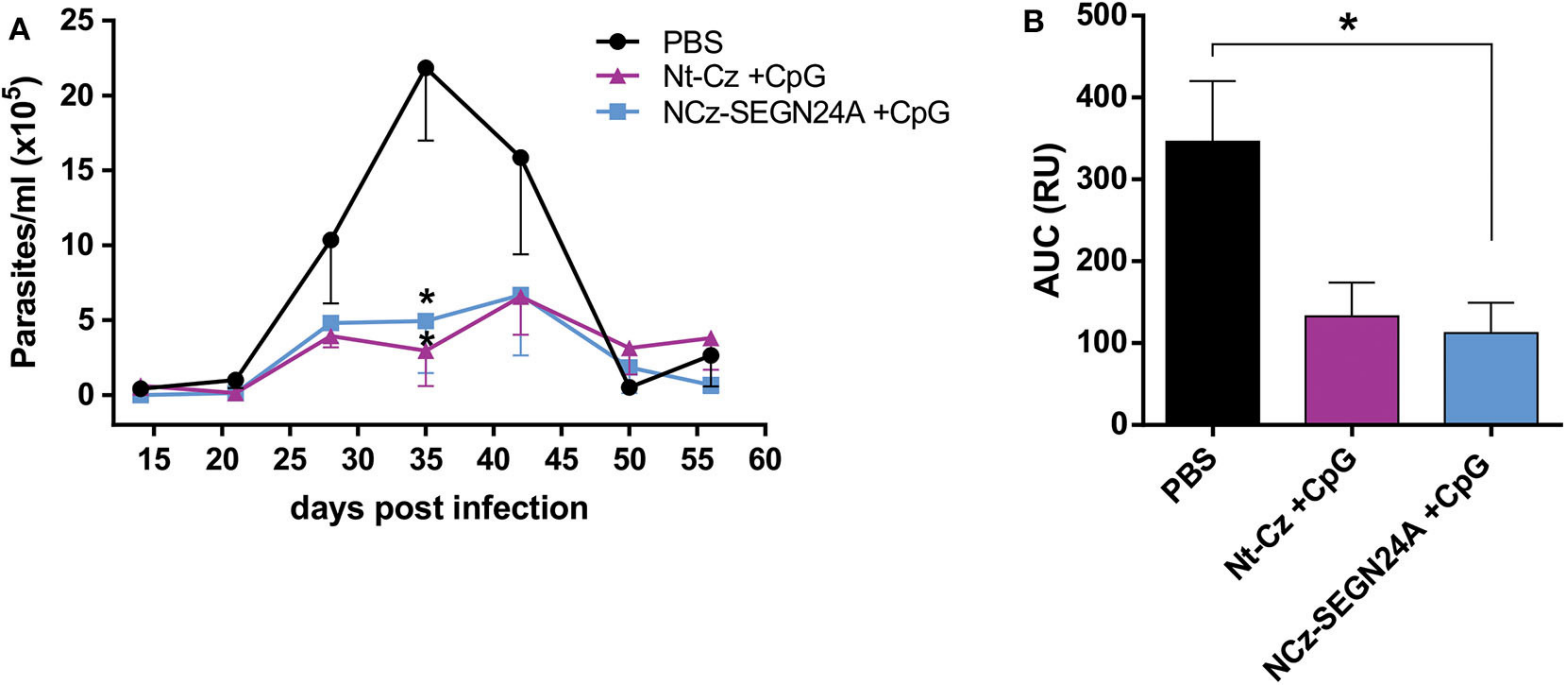
NCz-SEGN24A improves protection conferred by the single Nt-Cz antigen. Mice were either immunized with chimeric NCz-SEGN24A+CpG or with *T. cruzi* antigen Nt-Cz+CpG intramuscularly. Fifteen days after the last dose, mice were challenged with 3 × 10^5^ bloodstream trypomastigotes of K98 clone. **(A)** Parasitemia after infection was monitored weekly during its acute phase. **(B)** AUC of the parasitemia curve. Data are expressed as the mean ± SEM of two independent experiments. Asterisk represents statistical significance respect to saline group; *p < 0.05, and ns: non-significant difference. One-way ANOVA plus Tukey's post-test **(B)**.

After the last dose, animals were challenged with blood trypomastigotes of K98 clone, which belongs to a different DTU, TcI. On the acute phase of the infection, mice of both groups presented much lower amounts of circulating parasites (Figure 6A); at the peak of parasitemia (35 dpi), there were significant differences on the number of parasites for both groups against control (*p* < 0.05). However, when analyzing the areas under the total parasitemia curve, only mice immunized with NCz-SEGN24A+CpG presented significant reductions against control group in the total parasite loads (*p* < 0.05), with a 3-fold reduction of AUC (Figure 6B). Even though there was not a significant difference between AUC of Nt-Cz and NCz-SEGN24A groups, these results suggest an improvement in protection of the chimeric antigen vaccination over immunization with the single Nt-Cz antigen, which would be due to the inclusion of the superantigen domain in the chimera design.

## Discussion

Vaccination has proven to be the most successful strategy to stimulate systemic immune responses protecting against infection (42, 43). However, up to date, there are still many diseases for which there are no effective vaccines available. Among them, parasitic infections and particularly Chagas disease does not count yet with a vaccine generating sterilizing immunity (44).

In this work, with the aim to elicit a protective differential immune response against *T. cruzi*, we described a novel immunogen for vaccination purposes. We used a chimeric molecule that included a detoxified bacterial superantigen, which lacks its ability to bind to the TCR but retains the interaction with APCs, and an immunogenic domain of the *T. cruzi* antigen Cz. Thus, by mutation of one residue of major energetic contribution to TCR binding site (29), we obtained SEGN24A which showed non-significant increase in the frequency of mVβ8.2-bearing T cells *in-vivo*. In addition, by performing SPR assays, we observed a complete lack of interaction between SEGN24A and mVβ8.2. These results are in line with other authors' publications, where removal of the ability of different SAgs to bind the TCR alone is enough to ablate the recruitment of T cells bearing particular Vβ TCRs, and thus, cell proliferative activities responsible for SAgs toxicity (45, 46).

Although SEGN24A lost its ability to stimulate T cells, as it was expected, it retained its capability to interact with macrophages similarly as SEGwt did. In accordance with this, SEGN24A would be able to promote a proinflammatory profile, which could determine a Th1 environment without the elimination of the T cell effective pool. With the aim to determine if SEGN24A induced a M1 profile when interacting with macrophagic cells, we performed assays using the murine cell line RAW 264.7. We observed that, both wild-type and mutant SEG reduced the proliferation rate of a macrophagic cell line and significantly stimulated nitrites secretion on the supernatant of these cells, characteristic of classical macrophagic activation. These results agree with previous publications (26), where different non-classical SAgs, including SEG, inhibited proliferation of human THP-1 cell line differentiated to macrophages, associated with cell death. As it was stated before, cell death could result in DAMPs release, favoring immune response stimulation, as it was previously described for other adjuvants (36, 37). Noli Truant et. al. (26) also reported that SAgs promoted on macrophages a proinflammatory cytokines response of M1 profile that also included nitrites production. Our results suggest that the mutant SEGN24A maintains the MHC-II binding capacity, activating biological responses on macrophages, though in a lesser extent compared to SEGwt. In a previous work, we demonstrated that SEG does not interfere with the viability of murine DCs (27) and the present results allow us to hypothesize that SEGwt and SEGN24A display similar behavior with mouse DCs, which needs to be further confirmed.

In the design of the heterologous chimeric construct were included the bacterial mutant SAg SEGN24A and the N-terminal domain of cruzipain (Cz), the major cysteine protease of *T. cruzi*. Our choice was based on previous publications of our group, where we demonstrated that Nt-Cz is the catalytic and protective domain of the enzyme. During natural infection with the parasite, C-terminal domain of Cz is immunodominant, eliciting high titers of specific antibodies. When immunizations are performed with the full-length Cz, immune responses are mostly directed against the C-terminal domain, which is poorly protective. In contrast, immunization with Nt-Cz redirects the immune response to the protective domain, avoiding distraction of the immune system, thereby enhancing protection (17). Other chimeric molecules have already been designed including the Nt-Cz domain, showing very promising results in terms of protection conferred against *T. cruzi* infection (18, 19).

As a proof of concept, the heterologous chimeric antigen NCz-SEGN24A was tested with different immunization protocols. First, we analyzed whether NCz-SEGN24A has intrinsic immunogenic properties. By immunizing mice with NCz-SEGN24A without the addition of adjuvants, systemic-antibody titers were obtained against the parasite antigen. However, for a parasite homologous chimeric immunogen that also included Nt-Cz, Traspain, no specific antibodies responses were evoked without the addition of adjuvants (Sanchez Alberti, unpublished results). Moreover, the profile of elicited antibodies in NCz-SEGN24A immunized mice indicated higher IgG2a than IgG1 specific profiles, indicating that the immune response was predominantly oriented by the Th1 subset, which is desirable in the design of vaccines against intracellular pathogens. Modest cellular responses were also elicited. These results suggest that NCz-SEGN24A could have self-adjuvanting properties which could be mediated by direct targeting to MHC-II on APCs, conferred by the superantigenic domain. Previous reports are in line with these findings, since conjugation of antigens to modified-superantigens promotes targeting to APCs by MHC-II binding and this improves antigen-specific immune responses (23, 46, 47). Nevertheless, further studies should be performed with NCz-SEGN24A, in order to prove this hypothesis.

The humoral and cellular responses triggered by NCz-SEGN24A immunization were translated into a tendency of reduction on parasite loads throughout the acute phase of the infection, after challenging mice with *T. cruzi* trypomastigotes. NCz-SEGN24A-immunized mice showed significant differences at the peak of parasitemia (35 dpi) compared to control, although the area under the total parasitemia curve (AUC) did not evidence statistical differences. Data points out that humoral responses toward the parasitic antigen are primed by immunization with NCz-SEGN24A alone. Nonetheless, since stronger cellular inductions are needed to control *T. cruzi* infection, the addition of adjuvants improves these responses.

Unmethylated synthetic CpG-ODNs have been widely used as adjuvants to improve humoral and cellular responses toward vaccine antigens, evidenced by several preclinical and clinical trials (48–50). In the current study, the inclusion of CpG-ODN potentiated humoral responses by inducing a ten-fold increase in IgG anti-Nt-Cz specific titers in mice immunized with NCz-SEGN24A; similar responses were obtained in mice immunized with both Nt-Cz and SEGN24A proteins. In addition, these immunizations displayed an IgG2a predominating isotype, accordingly with a Th1-oriented profile, and cellular responses were augmented when compared to adjuvantless NCz-SEGN24A immunization. Similar patterns of responses have been consistently reported by utilizing CpG-ODN for other antigens, including *T. cruzi* (13, 17, 35, 51–53). More importantly, sera samples from NCz-SEGN24A immunized mice were able to dramatically reduce the infection of Vero cells, compared with samples from PBS-vaccinated animals. These findings are in correspondence with former data showing highly significant inhibition of cell-invasion by sera from Nt-Cz+CpG immunized mice, whereas immunization with the C-terminal domain of Cz did not elicit a detectable inhibition (17). Thus, it could be inferred that subdominant B-cell epitopes capable of developing a high neutralizing activity are indeed retained in the chimeric NCz-SEGN24A protein. The promising results obtained using NCz-SEGN24A+CpG prompted us to analyze the cellular immune response induced by this chimeric immunogen. Preliminary results, obtained with four immunizations of NCz-SEGN24A+CpG, showed production of intracellular cytokines by CD4^+^ T cells. We observed differential intracellular levels of IFN-γ, TNF-α and IL-2 when compared with the control group immunized with PBS (unpublished results). Even though no specific CD8^+^ T cells activity was measured in this manuscript, a rich microenvironment in IL-2 would facilitate CD8^+^ T cell proliferation which would be essential to eradicate the infected cells. Since the dysfunction of the CD8^+^ T cell is a key to the persistence of *T. cruzi* in the murine model (54), functional CD8^+^ T cells (55, 56) could contribute to control the parasitemia in the chronic phase of the disease. Preliminary results carried out during the chronic phase of the disease suggest that mice immunized with four doses of NCz-SEGN24A+CpG displayed an undetectable parasitemia, determined by quantitative real time PCR (unpublished data).

After *T. cruzi* challenge, both immunization groups reduced the parasite loads throughout the acute phase of the infection. Nonetheless, mice vaccinated with the chimeric antigen conferred significant differences against control when comparing the areas under the total parasitemia curve. NCz-SEGN24A-immunized mice were also able to control parasitemia in the early days of the infection (9 dpi), which would be of particular relevance given the silent entry of the parasite and the consequent delayed generation of proper immune responses that allow the establishment of the infection (2, 57). Additionally, the NCz-SEGN24A-immunized group maintained 100% survival until 75 dpi, showing a better performance in protection than Nt-Cz plus SEGN24A group. We hypothesize that one of the reasons could be related to NCz-SEGN24A differential intracellular pathway inside APCs, as it was previously shown for SEGwt (27), which would allow longer times of exposure of the parasitic antigen to T cells. Importantly, when immunizations were performed with the individual antigens, the molar doses of Nt-Cz and SEGN24A were higher than those in chimeric NCz-SEGN24A (10 μg of total protein were administered in each protocol), meaning that vaccination with NCz-SEGN24A+CpG was more efficient at controlling parasitemia with lower amount of molecules of the specific parasite antigen.

When we compared NCz-SEGN24A+CpG immunization vs. Nt-Cz+CpG, by performing a challenge with trypomastigotes from a different strain and DTU than the one we had used in the former experiment, NCz-SEGN24A was the only group to show statistical differences in AUC against control. The chimeric antigen showed a clear protection in the assay performed, meaning that the inclusion of the SAg domain could indeed result advantageous. Furthermore, NCz-SEGN24A was able to show a significant protection with lower molar doses of the Nt-Cz antigen, considering that in fact in the Nt-Cz+CpG group the molar dose was even double than in Nt-Cz+SEGN24A+CpG group.

As a general conclusion, we were able to develop a novel heterologous immunogen including a bacterial superantigen which proved to be protective during the acute phase of the infection against *T. cruzi*. Protection was even extended toward *T. cruzi* strains belonging to different DTUs, although further analysis should be performed to better characterize immune response evoked by NCz-SEGN24A antigen as well as extension of protection during the chronic phase. Our results highlight the importance of developing chimeric molecules and multicomponent vaccines against *T. cruzi* infection, with the aim of reducing parasitemia from the first stages of the infection, and we strongly believe that inclusion of modified bacterial superantigens are very promising in this field.

## Data Availability Statement

The raw data supporting the conclusions of this article will be made available by the authors, without undue reservation.

## Ethics Statement

The animal experiments (antisera production) were performed in accordance with the School of Medicine, Animal Ethics Committee (University of Buenos Aires) CD resolution #: 3381-18 Authorities for Animal Health of IMPaM, School of Medicine.

## Author Contributions

MA, AS, AB, EM, and MMF conceived and designed experiments. MA, AS, DR, AB, MJF, LI, SN, and MS performed the experiments. MA, AS, EM, and MMF analyzed the data. MA, EM, and MMF wrote the paper. All authors contributed to the article and approved the submitted version.

## Conflict of Interest

The authors declare that the research was conducted in the absence of any commercial or financial relationships that could be construed as a potential conflict of interest.
