# Supplementary material for: Heterologous Chimeric Construct Comprising a Modified Bacterial Superantigen and a Cruzipain Domain Confers Protection Against Trypanosoma cruzi Infection
Source: Front Immunol. 2020 Jun 30;11:1279. doi: 10.3389/fimmu.2020.01279 (PMC7338481; doi:10.3389/fimmu.2020.01279)

Supplementary figure 1. Construction of *ncz-segn24a* fused gene by PCR. The *nt-cz* gene was amplified from pET-23a plasmid, the PCR product was separated by 1.5% agarose gel electrophoresis, and *nt-cz* product was purified using Silica Bead DNA Gel Extraction Kit (Thermo Fisher Scientific). **(A)** Purified *nt-cz* was used as a template of a second PCR, in which primers PF_1 and PR_1 were used, extending *nt-cz* sequence with 30 nucleotids (nts) of *segn24a* gene. The PCR product was purified and furthered used as a template in a third PCR with primers PF_1 and PR_2, extending *nt-cz* sequence with 60 nts of *segn24a* gene. This procedure was repeated once more, obtaining *nt-cz* gene extended in 90 nts with *segn24a* sequence. **(B)** In another PCR, *segn24a* gene was amplified from pET-26b recombinant plasmid using primers PF_2 and PR_4 and the PCR product was purified from an agarose gel. **(C)** To fuse *segn24a* to the 3' region of *nt-cz*, PCR purified products of **A** and **B** were used as templates and primers PF_1 and PR_4 were annealed and extended by SOE-PCR. The resultant fused gene was *ncz-segn24a*.

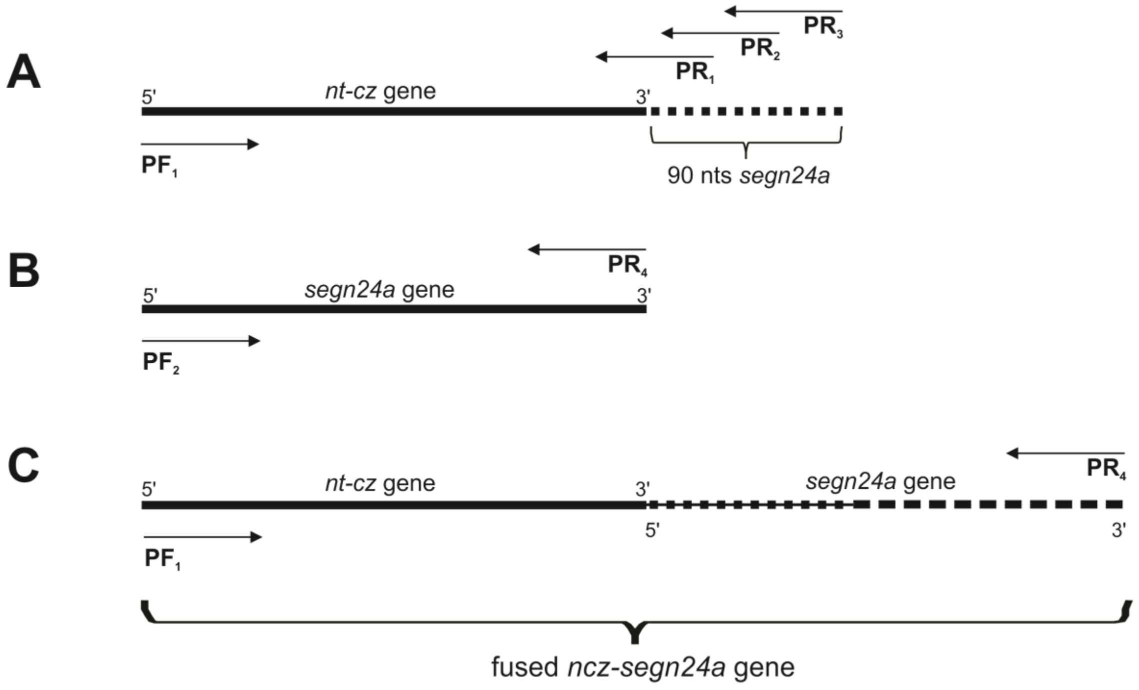

Supplement: Supplementary file 1 [file Data_Sheet_1.PDF]
